# Psychometric Properties of the Italian Adaptation of the Subjective Cognitive Reserve Questionnaire Based on Modifiable Lifestyle Factors (SCR-Q)

**DOI:** 10.3390/healthcare14070924

**Published:** 2026-04-02

**Authors:** Elisa Fabrizi, Carmen Moret-Tatay, Chiara Terribili, Gianluca di Cicco, Gabriele Aversa, José María Tormos-Muñoz, Francesco Della Gatta

**Affiliations:** 1 Doctoral School, Catholic University of Valencia San Vicente Mártir, 46002 Valencia, Spain; 2 National Center for Disease Prevention and Health Promotion, National Institute of Health, 00161 Rome, Italy; 3 Faculty of Psychology, Catholic University of Valencia San Vicente Mártir, 46100 Valencia, Spain; 4 ASL Roma 2, 00145 Rome, Italy; 5 Department of Neuroscience, Mental Health and Sense Organs (NESMOS), Faculty of Medicine and Psychology, Sapienza University of Rome, 00189 Rome, Italy; 6 Ospedale San Giovanni Battista—ACISMOM, 00148 Rome, Italy; 7 Facultad de Medicina y Ciencias de la Salud de la Universidad Católica de Valencia San Vicente, 46001 Valencia, Spain

**Keywords:** subjective cognitive reserve, general population, modifiable lifestyle factor

## Abstract

Background: As accelerating population aging increases the burden of neurological disorders, assessing cognitive reserve has become crucial for identifying resilience to decline. However, its evaluation is often hindered by instruments lacking the cultural sensitivity and psychometric rigor necessary for different populations. Objective: This study aimed to examine the psychometric properties and factorial structure of the Italian version of the Subjective Cognitive Reserve Questionnaire (SCR-Q) in a general adult population. Methods: A total of 225 adults (mean age = 51.84 years, SD = 14.08; 69.8% women) completed an online assessment including the SCR-Q and resilience, life satisfaction, and anxiety measures in an incidental sample selection. The sample was randomly divided for exploratory factor analysis (EFA) and confirmatory factor analysis (CFA). Internal consistency, factorial validity, and convergent/divergent validity were evaluated. Results: EFA and CFA supported a two-factor structure reflecting a physical health dimension and a cognitive–social dimension, with the two-factor model showing acceptable fit and outperforming a unidimensional solution. Internal consistency was adequate for the total scale (α = 0.795; ω = 0.850) and for both subfactors. SCR-Q scores were positively associated with resilience and life satisfaction and negatively associated with anxiety, with stronger associations observed for the cognitive–social factor. Conclusions: The Italian SCR-Q demonstrates satisfactory psychometric properties and supports a multidimensional conceptualization of subjective cognitive reserve integrating physical, cognitive, and psychosocial domains. The instrument represents a brief and ecologically valid tool for population-level screening and prevention research.

## 1. Introduction

The aging of the population represents one of the most relevant health and social challenges. According to demographic projections, the number of people aged 65 years or more will double, rising from 761 million in 2021 to 1.6 billion in 2050 worldwide [1]. This significant increase in the older population will also lead to a consequent higher incidence of age-related neurological diseases.

In this scenario, scientific research is increasingly focusing on potential protective factors that can prevent or delay cognitive decline. Among these, the concept of cognitive reserve has gathered more attention and relevance in the field of neuroscience and neuropsychology. The cognitive reserve construct refers to the adaptability of cognitive processes and it is defined as “a property of the brain that allows for cognitive performance that is better than expected given the degree of life-course related brain changes and brain injury or disease” [2].

To provide conceptual clarity, it is essential to distinguish cognitive reserve from other interrelated constructs. Unlike structural brain reserve, which refers to quantitative parameters of a neural substrate, cognitive reserve represents an active functional process [2,3]. Furthermore, cognitive reserve must be differentiated from cognitive capital, which encompasses the relational networks enabling individuals to acquire knowledge and adaptively respond to environmental risks throughout the life course, and environmental enrichment, which is defined as the multidimensional external setting that encourages positive stimulation and interaction [4,5]. Within this framework, neuroplasticity acts as the underlying biological mechanism that enables cognitive reserve to translate into maintained psychological well-being and cognitive efficiency.

Studies have shown how higher levels of cognitive reserve are associated with higher cognitive resilience and with the delay of neurodegenerative disease onset [6,7]. However, this preventive scope should not be considered determinative. The precise predictive validity of cognitive reserve remains an area of active investigation, as standardized criteria to quantify how life experiences translate into specific delay intervals have yet to be fully established.

Despite the fact that scientific interest in this topic is increasing, the evaluation of cognitive reserve still represents a challenge. Several instruments have been investigated to indirectly estimate cognitive reserve, mainly through self-administered questionnaires, with most based on schooling [8]. Controversies persist regarding the operationalization of cognitive reserve through self-reports primarily due to potential recall bias. Nevertheless, these tools may act as critical subjective indicators that complement objective measures by capturing the lifelong trajectory of life experiences that cross-sectional objective data may overlook.

Furthermore, these instruments may present some limitations when they are applied to a population other than the one for which they were initially created, such as the lack of a proper psychometric evaluation, cultural adaption and standardization to the general population.

This study aims to develop a psychometrically robust adaptation of the Subjective Cognitive Reserve Questionnaire (SCR-Q) for application within the Italian sociodemographic and cultural context, addressing the inherent limitations of the direct translation of assessment instruments [9]. The proposed adaptation involves a rigorous analysis of psychometric properties, including internal consistency and convergent validity, to ensure its scientific utility in the Italian population [10]. Such efforts are particularly relevant in light of ongoing demographic changes, as the proportion of individuals aged over 65 in Italy is projected to reach approximately 35% by 2045–2050, highlighting the urgent need for culturally appropriate and validated tools to assess cognitive reserve in aging populations [11].

Existing cognitive reserve instruments, although widely used, frequently show limitations when assessing life experience-based constructs that vary substantially across sociocultural contexts [12,13]. Developing a context-specific instrument is therefore essential to improve the accuracy of subjective cognitive reserve estimation and to support targeted interventions aimed at delaying age-related neurological disorders in Italy [13]. In this regard, the adapted SCR-Q is expected to provide a reliable method for estimating subjective cognitive reserve while acknowledging that, although cognitive reserve is shaped by lifelong activities, its assessment indices may differ across age groups and individuals [14]. Furthermore, the adaptation of the tool from the Spanish to the Italian context was not limited to linguistic translation but also sought to achieve rigorous semantic and metric equivalence. There are international gaps in the availability of brief and validated tools for the middle-aged Mediterranean population. This study tries to fill this gap by providing a psychometric link between Spanish and Italian gerontological research, allowing cross-cultural comparisons on the stability of the reserve construct in socio-cultural contexts that are similar but distinct in terms of lifestyle and education systems.

Accordingly, the present framework is grounded in established cognitive reserve models and incorporates Italian cultural specificities, integrating the concept of lifelong neuroplasticity and recognizing cognitive reserve as a dynamic protective factor influencing the clinical expression of neurological conditions [14]. It also addresses practical limitations of current instruments, such as administration time, and seeks to identify the most salient modifiable lifestyle factors within the Italian context to enhance ecological validity [15]. Importantly, the adapted instrument conceptualizes subjective cognitive reserve through multidimensional self-perceived domains that reflect everyday functioning and health-related behaviors. These domains include perceived physical condition, overall health status, dietary quality, general cognitive capacity, sleep hygiene, personal goals and life projects, socialization and perceived social support, and motivation to learn new things. Together, these elements capture the experiential and behavioral substrate through which individuals interpret subtle cognitive changes in daily life, thereby linking subjective cognitive decline with modifiable lifestyle and psychosocial determinants of cognitive reserve.

This multidimensional perspective aligns with evidence indicating that education, often used as a proxy of cognitive reserve, should be considered alongside occupational attainment, leisure engagement, and lifelong learning attitudes as equally meaningful contributors to subjective cognitive reserve [16,17]. The integration of the SCR-Q into Italian clinical practice may therefore improve the early detection of subjective cognitive decline, enabling the differentiation between normative aging and pathological processes and supporting timely referral for comprehensive neuropsychological evaluation and individualized care planning [18].

## 2. Materials and Methods

### 2.1. Participants

Participants were recruited through a multi-channel convenience sampling strategy. This study was disseminated via institutional channels at the Sant’Andrea Hospital in Rome, as well as through community-based outreach and professional networks to ensure a diverse range of respondents. Data were collected between June 2025 and January 2026.

Criteria for inclusion were being an adult aged between 18 and 85 years old, being fluent in the language in which the study was conducted, and giving informed consent to participate in the study. A total of 225 participants were included in the database. The mean age was 51.84 years (SD = 14.08), with an age range of 18–82 years. The sample was mainly composed of women (69.8%, n = 157), whereas men accounted for 30.2% (n = 68).

The sample was predominantly highly educated, with 82.7% reporting more than 13 years of education, whereas 14.2% reported ≤ 13 years, 2.2% ≤ 8 years, and 0.9% ≤ 5 years. Participants most frequently resided in Rome (34.7%, n = 78), while the remaining respondents were distributed across numerous other cities, each typically representing a small proportion of the sample (e.g., Genova 2.2% and several cities around 1–2%).

Regarding marital status, the majority were married (57.8%), followed by single (21.3%), cohabiting (13.3%), divorced (7.1%), and widowed (0.4%). In terms of occupational status, the largest group consisted of full-time employees (54.2%), followed by retirees (18.2%), self-employed individuals (15.6%), part-time employees (6.7%), unemployed individuals (4.0%), and students (1.3%).

Finally, most respondents reported having no dependent children (54.2%), whereas the remainder reported one (24.0%), two (18.2%), three (3.1%), or four or more (0.4%). All participants completed the assessment battery through an online questionnaire. The full sample was then randomly split into two independent groups to conduct the exploratory factor analysis (EFA) and confirmatory factor analysis (CFA), respectively.

### 2.2. Procedure

Sociodemographic characteristics were collected through a questionnaire created for this study, together with other instruments described below. The questionnaire was completed online via Google Forms.

Subjective Cognitive Reserve Questionnaire (SCR-Q): This comprises 8 items evaluating perceptions about one’s nutrition, physical condition, sleep quality, cognitive function, willingness to learn, social interaction, overall health, and life planning. An Italian translation of the SCR-Q was developed through the back translation of the original version in Spanish [9], following established guidelines [19]. A bilingual researcher translated the scale, another performed the back translation, and all items were examined by the authors to ensure conceptual equivalence.

GAD-7: The GAD-7 is a unidimensional scale consisting of 7 items, designed to assess the presence of symptoms of generalized anxiety disorder. It is a self-administrated scale, with each item asking the respondent to rate the severity of their symptoms over the past two weeks. Response options range from 0 to 3, corresponding to “not at all”, several days”, “more than half the days”, and “nearly every day”. The GAD-7 has demonstrated good validity and reliability [20]. It showed Cronbach’s α = 0.869 and McDonald’s ωₜ = 0.900. The Italian version of the instrument has shown adequate validity and reliability [21].

Satisfaction with Life Scale (SLSW): This is a global measure of overall life satisfaction. It asks respondents to indicate their level of agreement with a set of statements on a 7-point Likert scale, where 1 represents “strongly disagree” and 7 represents “strongly agree” [22]. Scores are summed to yield a total ranging from 5 to 35, with higher scores reflecting greater life satisfaction. It demonstrated Cronbach’s α = 0.833 and McDonald’s ωₜ = 0.883 in the current sample. It has been demonstrated that the Italian version of the SLSW is a valid and reliable instrument in the Italian context [23].

Brief Resilience Coping Scale (BRCS): This resilience scale measures optimism, creativity, perseverance, and growth in times of adversity [24]. The authors describe it as a proactive way to solve problems. The BRCS is a proven instrument for measuring resilience with adequate levels of validity and reliability. In the current sample, Cronbach’s alpha = 0.746 and McDonald’s omega (total) = 0.842. The Italian version of the BRCS has proved to be useful in measuring resilience in the Italian population [25].

### 2.3. Data Analysis

IBM SPSS software v. 27 and Jasp 0.18.3.0 were used for the analysis of data. Sociodemographic characteristics were analyzed with descriptive statistics. The significant level was set at *p* ≤ 0.05 and aspects related to possible confounding variables (e.g., outliers and the normality of data) were reviewed.

### 2.4. Ethics

The data, also processed using electronic tools, will be disclosed only in a strictly anonymous and aggregated form, for example, through scientific publications, statistics and scientific conferences. If personal data are transferred to another country or an international organization, all the guarantees provided by the GDPR 679/2016 will be adopted. This study was approved by the Ethical Committee of the Sapienza University of Rome (protocol code: 6853).

## 3. Results

The results are organized into three complementary analytical stages. First, an exploratory factor analysis (EFA) was conducted to examine the underlying dimensional structure of the SCR-Q and to identify the number and composition of latent factors. Second, a confirmatory factor analysis (CFA) was performed to test and compare competing structural models derived from the EFA with those derived from the original theoretical proposal, allowing an evaluation of model fit and parameter estimates. Finally, convergent and divergent validity analyses were carried out through correlations with external psychological measures (resilience, life satisfaction, and anxiety) in order to determine whether the SCR-Q total score and its subfactors show theoretically consistent patterns of association. Internal consistency was adequate: Cronbach’s alpha = 0.795 and McDonald’s omega (total) = 0.850.

### 3.1. Exploratory Factor Analysis (EFA) n = 112

**Figure 1:**
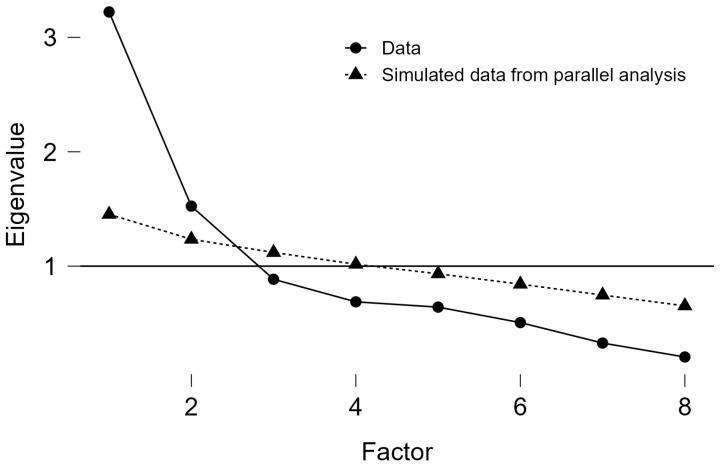
Parallel analysis for the SCR-Q scores in the EFA.

To assess the suitability of the correlation matrix for factor analytic procedures, we computed the Kaiser–Meyer–Olkin measure of sampling adequacy (KMO = 0.706; *p* < 0.001), Moreover, Barlett’s text χ^2^ (28) = 298.79. Mardia’s test indicated a significant violation of multivariate normality, with significant skewness (χ^2^(120) = 287.79, *p* < 0.001) and kurtosis (z = 8.13, *p* < 0.001), suggesting that the assumption of multivariate normality was not met. Therefore, a robust estimation method was employed in the CFA to account for non-normal data. As depicted in Figure 1, parallel analysis indicated that two factors should be retained, as only the first two observed eigenvalues exceeded those derived from simulated random data. Likewise, a visual inspection of the scree plot (Cattell’s criterion) suggested a two-factor solution, consistent with the parallel analysis. This two-factor model was subsequently compared with the original one-factor SCR-Q structure.

Table 1 displays the factor loadings for the Italian adaptation of the SCR-Q, revealing a two-factor solution with items related to physical and general health loading primarily on Factor 1 and items reflecting cognitive, social, and motivational aspects loading on Factor 2.

### 3.2. Confirmatory Factor Analysis (CFA) n = 113

A CFA using maximum likelihood estimation was conducted as a robust method to evaluate the factorial structure of the SCR-Q across one-factor versus two-factor solutions. The single-factor model showed poor overall fit to the data, χ^2^(20) = 72.49, *p* < 0.001. Incremental fit indices were below recommended thresholds (CFI = 0.727, TLI = 0.617, NFI = 0.670, IFI = 0.737, RFI = 0.539), and absolute fit indices also indicated substantial misfit (RMSEA = 0.152, 90% CI [0.115, 0.190], SRMR = 0.098). Information criteria values were AIC = 2319.01, BIC = 2384.68, and SSABIC = 2308.82. Collectively, these indices suggest that the unidimensional solution does not adequately reproduce the observed covariance structure. All standardized factor loadings were statistically significant (ps ≤ 0.008), ranging from 0.221 to 0.863. The strongest indicators were physical condition (λ = 0.863) and general health (λ = 0.626), whereas motivation to learn new things showed the weakest loading (λ = 0.221). Item explained variance (R^2^) ranged from 0.087 to 0.606, indicating heterogeneous contributions of indicators to the latent construct.

The proposed two-factor model showed an acceptable to good fit to the data, χ^2^(19) = 34.64, *p* = 0.015. Incremental fit indices were within or near recommended thresholds (CFI = 0.919, TLI = 0.880, IFI = 0.922, RNI = 0.919, NFI = 0.843), while absolute fit indices also supported adequate model fit (RMSEA = 0.085, 90% CI [0.037, 0.129], *p* = 0.101; SRMR = 0.070). Information criteria values (AIC = 2283.15; BIC = 2351.56; SSABIC = 2272.54) were lower than those observed for the one-factor model, indicating improved parsimony-adjusted fit. Additional indices (GFI = 0.996; MFI = 0.934; ECVI = 0.742) further supported the adequacy of the two-factor representation. As depicted in Table 2, factor loadings were adequate except for sleeping quality, which was retained for holistic reasons.

Pearson correlation analyses were carried out and showed that SCR-Q scores were positively associated with resilience (BRCS; *r* = 0.28, *p* = 0.002) and life satisfaction (SWL; *r* = 0.56, *p* < 0.001) and negatively associated with anxiety symptoms (GAD-7; *r* = −0.43, *p* < 0.001). Similarly, BRCS was positively correlated with SWL (*r* = 0.40, *p* < 0.001) and negatively correlated with GAD-7 (*r* = −0.28, *p* = 0.002), whereas SWL showed a moderate negative association with GAD-7 (*r* = −0.44, *p* < 0.001).

### 3.3. Convergency and Divergency Across Total Scores and Subfactors with BRCS, GAD-7 and SWLS

In Table 3, we examined Pearson correlations between the SCR-Q total score, its two subfactors (physical: SCR-F-Q; cognitive: SCR-C-Q), and external measures (BRCS, SWL, and GAD-7). The correlation pattern indicates that the BRCS aligns more closely with the cognitive subfactor than with the physical subfactor: the SCR-C-Q–BRCS association is positive and statistically significant, whereas the SCR-F-Q–BRCS association is weaker and not statistically significant. In contrast, both subfactors show the expected directional relationships with the other validation measures (positive with SWL and negative with GAD-7), and the two subfactors are moderately interrelated, suggesting related but distinguishable dimensions underlying the SCR-Q total score.

For the physical subfactor of SCR-Q, comprising dietary quality, physical condition, sleep hygiene, and general health, internal consistency ranged from acceptable to good, with Cronbach’s α = 0.756 and McDonald’s ωₜ = 0.848. For the second factor, including perceived general cognitive capacity, motivation to learn new things, socialization and social support, and personal goals and life planning, internal consistency was acceptable, with Cronbach’s α = 0.703 and McDonald’s ωₜ = 0.818. 

Lastly, differences across age groups (18–40, 41–65, >65) were not statistically significant. As shown in Table 4, mean scores were highly comparable across groups for the total SCR-Q (Young: 30.14; Adults: 31.86; Older Adults: 30.22) and for the SCR-P-Q (14.18, 15.10, 14.04) and SCR-C-Q subscales (15.96, 16.76, 16.17). Dispersion indices were also similar, with only a slightly lower coefficient of variation in the middle-aged group, suggesting modestly greater homogeneity but no meaningful differences in overall levels.

Importantly, these findings support the idea that cognitive reserve, particularly as captured through modifiable lifestyle-related factors, is not strictly age-dependent. Instead, it appears relatively stable across adulthood, reinforcing the notion that lifestyle behaviors (e.g., engagement, activity, health habits) contribute to cognitive reserve in a way that is not inherently determined by chronological age. This aligns with a lifespan perspective in which individuals can build and maintain cognitive reserve through ongoing, modifiable experiences rather than age alone.

## 4. Discussion

The present study examined the psychometric properties and factorial structure of the Italian adaptation of the Subjective Cognitive Reserve Questionnaire and explored its construct validity in a general adult population. The findings support the reliability and validity of the adapted instrument and provide evidence for a multidimensional representation of subjective cognitive reserve consistent with contemporary theoretical models of cognitive reserve and resilience in aging.

Both exploratory and confirmatory factor analyses supported a two-factor structure distinguishing the physical and general health dimension from the cognitive, motivational, and social dimension. This structure departs from earlier unidimensional operationalizations of cognitive reserve but is coherent with theoretical frameworks proposing that reserve emerges from the interaction between brain integrity, health behaviors, and psychosocial engagement across the lifespan. Foundational and contemporary models describe cognitive reserve as the adaptability of cognitive processes that allows individuals to cope better than expected with age-related brain changes or pathology, emphasizing the role of lifelong experiences and environmental enrichment in shaping resilience to decline. Converging epidemiological evidence shows that lifestyle and psychosocial factors, including physical health, nutrition, sleep, social participation, and cognitive engagement, contribute meaningfully to resilience against neuropathology and dementia risk [26,27,28,29,30,31].

Within the physical dimension, perceived physical condition, general health, nutrition, and sleep hygiene clustered coherently, consistent with research linking cardiometabolic health, lifestyle behaviors, and sleep quality to brain aging and cognitive trajectories [32,33]. In parallel, the cognitive and psychosocial dimension, comprising perceived cognitive capacity, socialization and support, life goals, and motivation for learning, reflects domains closely associated with adaptive functioning, purpose in life, and sustained cognitive engagement, all of which have been related to a reduced risk of cognitive impairment and dementia in longitudinal population studies [34,35,36]. Although sleep hygiene and motivation to learn showed comparatively weaker loadings, their theoretical relevance for neuroplasticity and lifelong stimulation supports their retention within a holistic construct of subjective reserve [37,38].

Construct validity analyses further reinforced the interpretability of the instrument. Positive associations with resilience and life satisfaction, alongside negative associations with anxiety, followed theoretically expected patterns and align with evidence linking psychological well-being, adaptive coping, and lower affective distress to better cognitive outcomes in community-dwelling adults [39,40,41]. These associations likely stem from greater flexibility in high-reserve individuals, facilitating more effective stress regulation. However, causality may be bidirectional: while reserve promotes resilience, chronic anxiety may act as a depleting factor that discourages the social and cognitive engagement necessary to accumulate reserve. The stronger association between resilience and the cognitive and psychosocial factor suggests that motivational and social engagement mechanisms may be central to subjective reserve, echoing population-based findings showing that social participation and cognitive activity contribute to maintained cognition across aging.

From a preventive perspective, the multidimensional structure observed here aligns with conceptualizations of subjective cognitive decline as an early self-perceived stage situated at the intersection of neurobiological vulnerability and psychosocial context. Instruments capable of capturing experiential, behavioral, and emotional domains may therefore improve early screening and risk stratification in community and primary care settings, where brief and ecologically valid tools are essential for large-scale monitoring and prevention.

Several limitations should be acknowledged. The cross-sectional design precludes conclusions regarding predictive validity or longitudinal sensitivity to cognitive decline. Reliance on self-report measures may introduce shared method variance and subjective bias. Regarding the representativeness of the findings, it should be acknowledged that the use of a convenience sample recruited through hospital and community networks may introduce sampling bias. Specifically, the sample may lean towards individuals with a higher interest in cognitive health or those with specific sociocultural backgrounds. Consequently, caution is advised when generalizing these results to the broader national population.

In addition, further research is needed to test measurement invariance across age, sex, and educational strata within the general population. Future studies should incorporate longitudinal follow-up, neuropsychological performance, and biological markers to evaluate predictive and criterion validity in line with current recommendations for advancing cognitive reserve research.

Despite these limitations, the findings provide initial empirical support for the Italian version of the questionnaire as a brief, psychometrically sound, and theoretically grounded tool suitable for the general population.

## 5. Conclusions

The Italian adaptation of the Subjective Cognitive Reserve Questionnaire shows adequate psychometric properties and supports a two-factor structure reflecting physical health and cognitive–social dimensions of subjective cognitive reserve in the general population. This multidimensional organization is consistent with contemporary models of cognitive reserve that integrate lifestyle, psychosocial engagement, and perceived cognitive functioning as complementary contributors to resilience in aging. The SCR-Q therefore represents a brief and ecologically valid tool for population-level screening and prevention research. Future longitudinal studies are needed to confirm its predictive value for cognitive decline and clinical outcomes.

## Figures and Tables

**Table 1:** Factor Loadings for the Italian adaptation of SCR-Q.

	Factor 1	Factor 2	Uniqueness
La sua condizione fisica	**0.986**	0.107	0.017
La sua salute generale	**0.645**	0.361	0.454
La qualità della sua alimentazione	**0.625**	0.045	0.608
La sua capacità cognitiva generale	0.288	**0.613**	0.541
La sua igiene del sonno	0.286	0.283	0.838
I suoi obiettivi e il suo progetto di vita	0.282	**0.454**	0.715
La sua socializzazione e il supporto sociale	0.067	**0.742**	0.445
La sua voglia di imparare cose nuove	0.013	**0.728**	0.470

Note. In bold, higher factor loadings marked. Note: Items were translated into Italian following a forward–backward translation procedure. Two bilingual experts independently translated the original items into Italian, and discrepancies were resolved by consensus. A separate bilingual translator, blind to the original version, performed the back translation into English. The final wording was reviewed by the research team to ensure semantic and conceptual equivalence. The English versions are provided here for reference: “The quality of your nutrition”, “Your physical condition”, “Your sleep hygiene”, “Your overall cognitive ability”, “Your willingness to learn new things”, “Your socialization and social support”, “Your overall health”, and “Your goals and vital plan.”

**Table 2:** Factor loadings for CFA in two-factor solution.

						95% Confidence Interval	
Factor	Indicator	Estimate	Std. Error	z-Value	*p*	Lower	Upper
Factor 1	*La qualità della sua alimentazione* The quality of your nutrition	0.660	0.099	6.648	<0.001	0.465	0.855
	*La sua condizione fisica* Your physical condition	0.995	0.102	9.804	<0.001	0.796	1.194
	*La sua igiene del sonno*	0.321	0.099	3.256	0.001	0.128	0.515
	Your sleep hygiene *La sua salute generale* Your overall health.	0.581	0.078	7.417	<0.001	0.428	0.735
Factor 2	*La sua capacità cognitiva generale* Your overall cognitive ability	0.585	0.092	6.382	<0.001	0.405	0.764
	*La sua voglia di imparare cose nuove* Your willingness to learn new things	0.420	0.079	5.317	<0.001	0.265	0.575
	*La sua socializzazione e il supporto sociale* Your socialization and social support.	0.517	0.090	5.751	<0.001	0.341	0.693
	*I suoi obiettivi e il suo progetto di vita* Your goals and Vital plan	0.421	0.097	4.331	<0.001	0.231	0.612

**Table 3:** Pearson’s correlations across SCR-Q total score and subfactors.

			Pearson’s r		*p*	Lower 95% CI	Upper 95% CI
SCR-Q	-	SCR-P-Q	0.868	***	<0.001	0.814	0.907
SCR-Q	-	SCR-C-Q	0.786	***	<0.001	0.704	0.847
SCR-Q	-	BRCS	0.281	**	0.002	0.103	0.442
SCR-Q	-	SWL	0.555	***	<0.001	0.413	0.670
SCR-Q	-	GAD-7	−0.430	***	<0.001	−0.569	−0.267
SCR-P-Q	-	SCR-C-Q	0.375	***	<0.001	0.205	0.522
SCR-P-Q	-	BRCS	0.152		0.106	−0.033	0.327
SCR-P-Q	-	SWL	0.435	***	<0.001	0.272	0.573
SCR-P-Q	-	GAD-7	−0.330	***	<0.001	−0.484	−0.155
SCR-C-Q	-	BRCS	0.336	***	<0.001	0.162	0.489
SCR-C-Q	-	SWL	0.494	***	<0.001	0.341	0.621
SCR-C-Q	-	GAD-7	−0.392	***	<0.001	−0.537	−0.224
BRCS	-	SWL	0.397	***	<0.001	0.230	0.542
BRCS	-	GAD-7	−0.284	**	0.002	−0.445	−0.106
SWL	-	GAD-7	−0.437	***	<0.001	−0.575	−0.275

** *p* < 0.01, *** *p* < 0.001.

**Table 4:** Descriptives across age groups in SCR-Q total score and subfactors. SCR-Q total scores.

	N	Mean	SD	Coefficient of Variation
Young	28	30.143	5.233	0.174
Adults	63	31.857	3.542	0.111
Older Adults	23	30.217	5.359	0.177
Descriptives—SCR-P-Q				
Young	28	14.179	3.411	0.241
Adults	63	15.095	2.513	0.166
Older Adults	23	14.043	3.431	0.244
Descriptives—SCR-C-Q				
Young	28	15.964	2.963	0.186
Adults	63	16.762	1.949	0.116
Older Adults	23	16.174	2.640	0.163

## Data Availability

The datasets presented in this article are not readily available because they are part of a PhD research project. They will become available once the PhD is finished.

## Abbreviations

The following abbreviations are used in this manuscript:

SCR-Q	Subjective Cognitive Reserve Questionnaire
EFA	Exploratory factor analysis
CFA	Confirmatory factor analysis
SLSW	Satisfaction with Life Scale
BRCS	Brief Resilience Coping Scale
